# Xq22 deletion involving *TCEAL1* in a female patient with early-onset neurological disease trait

**DOI:** 10.1038/s41439-024-00278-9

**Published:** 2024-05-15

**Authors:** Keiko Shimojima Yamamoto, Yusuke Itagaki, Kazuki Tanaka, Nobuhiko Okamoto, Toshiyuki Yamamoto

**Affiliations:** 1 Department of Transfusion Medicine and Cell Processing, Tokyo Women’s Medical University, Tokyo, Japan; 2 Institute of Medical Genetics, Tokyo Women’s Medical University, Tokyo, Japan; 3 Department of Pediatrics, Suita Municipal Hospital, Suita, Japan; 4 Department of Medical Genetics, Osaka Women’s and Children’s Hospital, Izumi, Japan

**Keywords:** Cytogenetics, Genetics research

## Abstract

A 3.5-Mb microdeletion in Xq22 was identified in a female patient with early-onset neurological disease trait (EONDT). The patient exhibited developmental delay but no hypomyelination despite *PLP1* involvement in the deletion. However, the clinical features of the patient were consistent with those of *TCEAL1* loss-of-function syndrome. The breakpoint junction was analyzed using long-read sequencing, and blunt-end fusion was confirmed.

The Xq22 chromosomal region has attracted the attention of researchers because it contains the proteolipid protein 1 (*PLP1*) gene^1^. Duplication of the Xq22 region, including *PLP1*, is known to be the major genetic cause of Pelizaeus-Merzbacher disease (PMD; MIM# 312080), an X-linked hypomyelinating leukodystrophy characterized by impaired myelination of the central nervous system^2^. Patients with PMD are typically male and exhibit motor delay, nystagmus, spastic quadriplegia and ataxia. Hypomyelination can be detected in PMD patients using brain magnetic resonance imaging (MRI)^3^.

Compared with an increase in the genomic copy number of *PLP1*, the loss of the *PLP1* allele causes a milder phenotype of spastic paraplegia^4,5^. Because the symptoms are mild, cell survival during early embryonic development is not impaired. Therefore, X-chromosome inactivation (XCI) would not be triggered in women with small deletions restricted to the *PLP1* region, and mild spastic quadriplegia appears even in female carriers. However, larger Xq22 deletions in the *PLP1* region have different results.

In 2014, Xq22 deletions in females were reported to be associated with severe neurodevelopmental disorders^6^. This clinical entity was later described as early-onset neurological disease trait (EONDT), and additional cases have been reported^7^. Patients with EONDT are characterized by the onset of severe developmental delay/intellectual disability (DD/ID) in infancy, behavioral abnormalities, hypotonia, strabismus, and craniofacial dysmorphology, in which a potentially recognizable pattern is emerging. We recently encountered a female patient with EONDT. A detailed genotype–phenotype correlation was performed to further understand this disease entity.

A 5-year-old girl, who was born at 37 weeks and 6 days of gestation with a birth weight of 2465 g (10th~25th percentile), length of 48.5 cm (mean), and occipitofrontal circumference (OFC) of 34.3 cm (75th~90th percentile), presented with distinctive features, including a triangular face with a wide nasal bridge, widely spaced eyes, a long philtrum, and a prominent jaw (Fig. 1a). Her development was delayed from early infancy, achieving head control at nine months and rolling over at four years of age. Currently, she can sit with support but cannot stand. The patient exhibited no extrapyramidal signs, nystagmus, or digestive tract symptoms. No behavioral abnormalities, such as insomnia or self-injury, were observed. Her height, weight, and OFC at examination were 104 cm (25th~50th percentile), 16.4 kg (25th~50th percentile), and 49.3 cm (25th~50th percentile), respectively. Brain MRI revealed no definite abnormalities (Fig. 1a). We obtained parental consent for the publication of this case, including the patient’s detailed medical history and photographs.

**Fig. 1:**
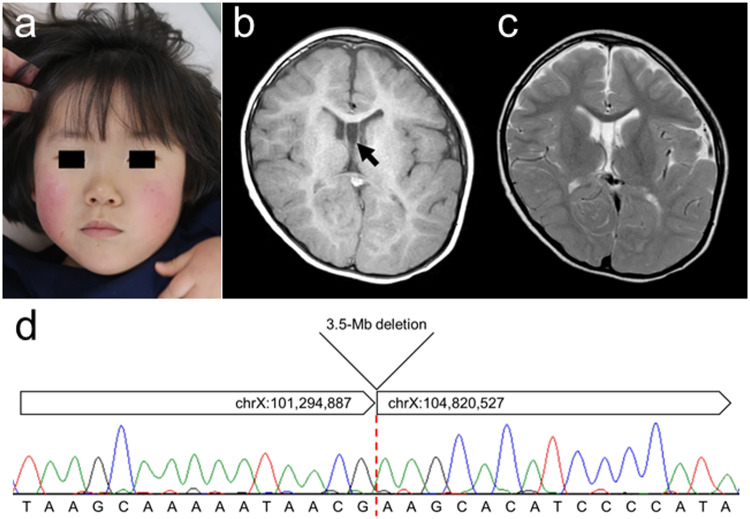
Details of the present patient. **a** Distinctive facial features, including a triangular face with a wide nasal bridge, widely spaced eyes, a long philtrum, and a prominent jaw, of the patient are noted. Written informed consent was obtained from her guardian for use of this photo. Results of brain MRI at 4 years. T1-weighted (**b**) and T2-weighted (**c**) axial images are shown. No hypomyelination is present, although cavum vergae can be observed (arrow). **d** Electropherogram of Sanger sequencing for the breakpoint junction. A 3.5-Mb deletion is confirmed, and there is no homologous sequence between the two breakpoints.

This study was conducted according to the Declaration of Helsinki, and the requisite permission was obtained from the institutional ethics committee. Peripheral blood samples were collected with written informed consent from the participant’s guardians. Chromosomal microarray testing was performed for first-tier genetic screening, which showed a loss of genomic copy number in the Xq22 region (arr[GRCh37] Xq22.1q22.3(101299765_104811259)×1). This region contained *PLP1*. To confirm the breakpoint of this aberration, long-read whole-genome sequencing was performed using nanopore sequencing as previously described^8^. Two breakpoints were detected by visual inspection using Integrative Genomics Viewer (IGV; https://igv.org/). The primer pair 5’-CTTATTACTCAACTGAAAAC-3’ and 5’-TAGGGTCATGTAGGTGTTGC-3’ was designed to amplify the breakpoint junction. The PCR products amplified using these primers were sequenced using the Sanger method. Finally, the breakpoint junction was confirmed, and the precise breakpoints were determined to be chrX:101,294,887 and chrX:104,820,527, indicating a 3.5-Mb deletion. No homologous sequence was detected between the two breakpoints, and blunt-end fusion was confirmed (Fig. 1b). PCR using the same primers was performed on the DNA sample obtained from the patient’s mother; due to the divorce of the patient’s parents, we could not obtain the father’s cooperation. No amplicons were obtained in the mother’s sample, indicating that the mother was not a carrier of this deletion, and a de novo occurrence was suspected. To determine the origin of the deletion, linkage analysis was conducted employing STS markers within the deletion region. The haplotype of the remaining allele of the patient was consistent with that of her mother (data not shown). Therefore, although a sample from her father could not be obtained, the deletion was determined to have occurred in the paternally derived allele. X inactivation status was analyzed using HUMARA, as described previously^9^. The results showed an imbalanced inactivation ratio between the paternally and maternally derived alleles (36% vs. 64%; Supplementary Fig. 1); however, this ratio did not fulfill the criteria for a skewed pattern.

The patient exhibited severe developmental delay, hypotonia, and distinctive facial findings, including a triangular face with a wide nasal bridge, widely spaced eyes, a long philtrum, and a prominent jaw (Table 1). Although strabismus and abnormal behaviors were not observed, the penetrance of these findings is not complete, and we diagnosed her with EONDT^7^. The size and range of the identified deletions were typical of those previously reported in patients with EONDT (Fig. 2).

**Table 1:** Clinical features of the present patient.

	Present patient	Typical patients with EONDT
Gender	Female	Female
Deletion size	3.5 Mb	
Birth gestation	37 weeks	
Age	5 years	
Developmental delay/Intellectual disability	Severe	+
Behavioral abnormality	None	+
Eyes	No nystagmus, no strabismus	±
Brain MRI	No abnormality	Delayed myelination
Spasticity	None	−
Gastrointestinal	None	±
Facial dysmorphism	Typical	±

**Fig. 2:**
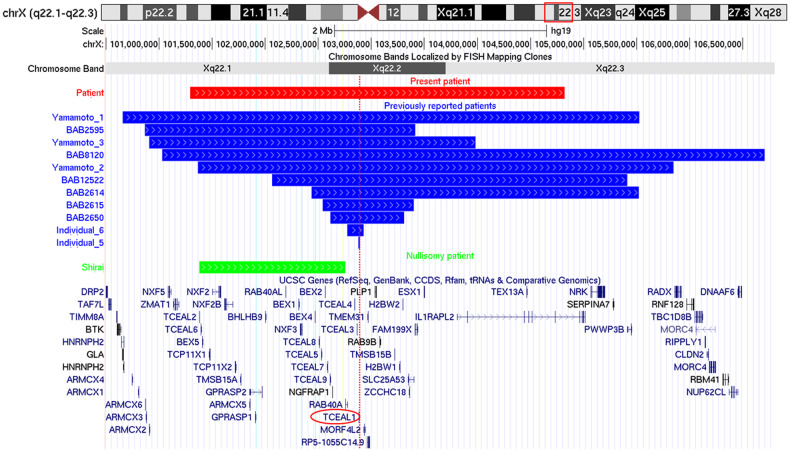
Genome map surrounding the deletion. The deletion identified in the present patient (red bar) is depicted with the previously reported deletions (blue bars) including *TCEAL1* (red circle). The dotted line indicates the position of *TCEAL1*. Yamamoto_1, 3, and 2 were reported by Yamamoto et al.^6^. BAB2595, 8120, 12522, 2614, 2615, and 2650 were reported by Hijazi et al.^7^. Individual_6 and 5 were reported by Hijazi et al.^10^.

Small deletions involving *PLP1* cause spastic paraplegia and delayed myelination, as observed by MRI, in both men and women. However, these small deletions are restricted to the 553-kb region of chrX:102,957,289_103,510,104^6^. In comparison, larger deletions including this region have not been observed in male patients manifesting with spastic paraplegia and are thought to be lethal in male embryos. The core features of EONDT, which can be considered a sex-influenced neurodevelopmental disease found only in females, are distinct in nature and are not parsimoniously explained by the inclusion of *PLP1* in the deletion region^7^. The identification of loss-of-function variants of the transcription elongation factor A (SII)-like 1 (*TCEAL1*) gene in patients with features overlapping with those of the EONDT phenotype in females with Xq22 microdeletions confirmed that this gene is responsible for this phenotype^10^. However, the exact reason why female patients with larger Xq22 microdeletions do not show abnormal findings on MRI or spastic paraplegia is still unclear. This absence is likely related to differences in the XCI status of the genes in the deletion region.

Most previously reported patients with EONDT exhibited skewed XCI^6,7^. Although the present patient did not fulfill the criteria for skewed XCI, the XCI pattern was not equal between the paternally and maternally derived alleles. If the normal allele is inactivated, the clinical features of spastic paraplegia associated with hypomyelination will occur. However, female embryos with large Xq22 deletions associated with predominant inactivation of the normal alleles cannot survive. There has been a case report of an Xq22 nullisomy in a male patient^11^. Although the deletion in the male patient did not include *PLP1* (Fig. 2), the patient exhibited extremely severe neurological impairment but no hypomyelination. This case indicates the existence of a neurologically important gene in the Xq22 region other than *PLP1*.

In 2022, *TCEAL1* was identified as the gene responsible for neurodevelopmental syndromes in both sexes^10^. Most of the identified nucleotide variants were nonsense alterations or truncations. Thus, loss of function (LoF) of this gene was considered the underlying mechanism. The clinical features of the patient included severe developmental delay, hypotonia, and abnormal behavior, which were compatible with those of EONDT patients. Based on these findings, chromosomal deletions involving *TCEAL1* were confirmed to be the genetic cause of EONDT. The clinical features of the present patient can also be attributed to the LoF or haploinsufficiency of *TCEAL1*.

Although *TCEAL1* was not included in the deletion region in the Xq22 nullisomy case mentioned earlier^11^, the clinical features of the male patient could be explained by *TCEAL1* LoF. Hence, it is plausible that the regulatory region of *TCEAL1* may be impacted by Xq22 deletion.

The junction features of Xq22 deletions have been reported by Hijazi et al.; explicit microhomology was observed in 10 of 13 patients (~77%), while insertional complexities were observed in 3 of 13 patients (~23%)^7^. These findings suggest end-joining mechanisms of double-strand break repair, nonhomologous end joining, and microhomology-mediated end joining. In the patient in this study, blunt-end fusion, believed to be a consequence of nonhomologous end joining, was detected, which is consistent with previous reports^12^.

## Supplementary information

Supplementary Figure S1

## Data Availability

The data that support the findings of this study are available from the corresponding author upon reasonable request.
