# Supplementary material for: Xq22 deletion involving TCEAL1 in a female patient with early-onset neurological disease trait
Source: Hum Genome Var. 2024 May 15;11:20. doi: 10.1038/s41439-024-00278-9 (PMC11096163; doi:10.1038/s41439-024-00278-9)
Supplement: Supplementary file 1 — Supplementary Figure S1 [file 41439_2024_278_MOESM1_ESM.pdf]

Supplementary Figure 1. The result of HUMARA assay

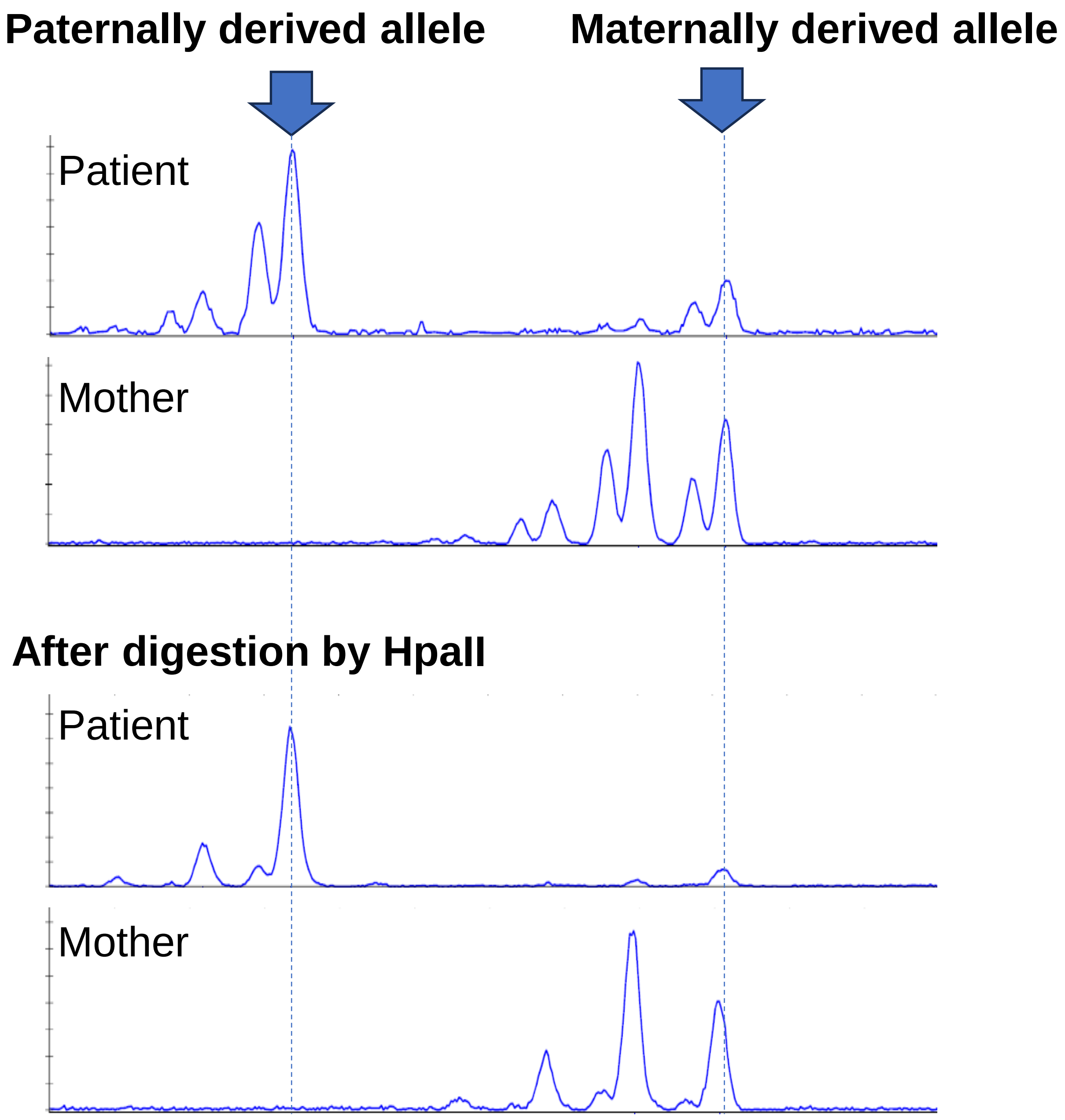

By HapII digestion, maternally derived allele is predominantly decreased. This indicates that paternally derived allele is predominantly inactivated.
